# Diethylnitrosamine induces lung adenocarcinoma in FVB/N mouse

**DOI:** 10.1186/s12885-018-4068-4

**Published:** 2018-02-07

**Authors:** Zsolt Mervai, Krisztina Egedi, Ilona Kovalszky, Kornélia Baghy

**Affiliations:** Department of Pathology and Experimental Cancer Research, Budapest, Hungary

**Keywords:** Lung cancer, NSCLC, Mouse model, Diethylnitrosamine, Tumorigenesis

## Abstract

**Background:**

Diethylnitrosamine is a well known carcinogen that induces cancers of various organs in mice and rats. Using FVB/N mouse strain, here we show that diethylnitrosamine induces primarily lung adenocarcinomas with modest tumor development in the liver, offering a new model to study chemical carcinogenesis in the lung.

**Methods:**

Animals were exposed to a single high dose of diethylnitrosamine, and more than 70% of the mice developed lung cancer. To obtain a new transplantable tumor line, pieces of primary tumors were inoculated and maintained subcutaneously in the same mouse strain. We used immunohistochemistry to characterize the tumor for main lung adenocarcinoma markers. We searched for mutations in KRAS exon 2 and EGFR exon 19, 21 with Sanger sequencing. We also compared the normal lung tissue with the diethylnitrosamine induced primary adenocarcinoma, and with the subcutaneously maintained adenocarcinoma using Western blot technique for main cell cycle markers and to identify the main pathways.

**Results:**

Primary and subcutaneous tumors express cytokeratin-7 and thyroid transcription factor-1, markers characteristic to lung adenocarcinoma. In addition, no mutations were found in the hot spot regions of KRAS and EGFR genes. We found high mTOR activation, but the level of p-Akt Ser473 and p-Akt Thr308 decreased in the tumorous samples.

**Conclusions:**

We established a new lung adenocarcinoma model using FVB/N mouse strain and diethylnitrosamine. We believe that this new model system would be highly useful in lung cancer research.

**Electronic supplementary material:**

The online version of this article (10.1186/s12885-018-4068-4) contains supplementary material, which is available to authorized users.

## Background

Cancer is the second leading cause of death nowadays [1, 2]. Lung cancer is the most frequent tumor all over the world which claims the most life among other cancer types in Europe and in the United States [1, 2]. According to their phenotype and clinical behavior lung cancers are divided to two major types: small cell lung cancer (SCLC) and non-small cell lung cancer (NSCLC). NSCLC is the more common type of lung carcinomas [3]. In the US 85% of the lung cancers are NSCLC [3]. In the last decade adenocarcinomas became the dominant representative within NSCLC [4]. These NSCLCs express proteins such as cytokeratin-7 (CK7) and thyroid transcription factor-1 (TTF1) which are diagnostic markers of the tumor [5, 6].

Lung adenocarcinomas belong to the first types of tumors where the importance of driver mutations has been discovered. So far treatment options are guided by the KRAS and the epidermal growth factor receptor (EGFR) status as the majority of mutations can be detected on KRAS exon 2 and EGFR exon 19, 21 [7–10]. EGFR signaling activates downstream pathways such as Akt/mTOR and MEK/Erk which then promote cell proliferation [11].

Because of the high representation of adenocarcinoma and its relative great number of targetable mutations compared to other cancer types it is one of the best examined cancer [3]. For in vitro studies cell lines are available, but for in vivo experiments the opportunities are limited. Lung cancer is inducible in mouse with Jaaksiegte sheep retrovirus, but only in the immunocompromised strains [12]. Benzopyrene and 4-(methylnitrosamino)-1-(3-pyridyl)-1-butanone induced lung carcinogenesis is a known and described way to create lung tumors, but only in few strains [13]. A Cre-recombinase mediated model also exists [14]. Here we present another easy way to develop lung adenocarcinoma in FVB/N mouse strain.

Diethylnitrosamine (DEN) is a well-known and widely used chemical compound to cause cancer in vivo [15]. The mechanism of action of DEN involves it’s adduct formation potential. After it’s bioactivation by CYP450 enzymes it transforms to be a strong alkylating agent that will form adducts in the DNA, which results in a direct carcinogen effect [16]. We injected FVB/N mice with one single dose of DEN. This lung carcinogen effect of DEN was described earlier in A/J mouse strain [17]. A/J strain is susceptible to lung cancer and after DEN exposure they developed lung adenocarcinomas which were positive for KRAS mutation in the 80% of the cases [17].

FVB/N mouse strain has also high susceptibility for lung cancer [18]. An aging study with FVB/N strain published in the literature indicated that lung and liver cancer were the two most represented tumor types developed. At age 14 months 14% of the population had lung cancers, but there were no liver tumors. The former increased to 38% in the 24 months old population but only 6% had liver cancer. The population contained both males and females [18].

Our primary aim was to assess the lung cancer initiation potency of DEN in FVB/N strain and also determine the KRAS and EGFR status of the tumors which could later serve as a new model for NSCLC research. We wanted to compare the characteristics of DEN induced and spontaneously developed lung tumors by their markers and molecular status. We also aimed to determine the main signaling pathways driving tumor proliferation together with the status of the cell cycle.

## Methods

### Animals and treatments

All animal experiments were conducted according to the ethical standards of the Animal Health Care and Control Institute Csongrád County, Hungary. The protocol was approved by the Committee of the Animal Health Care and Control Institute Csongrád County, Hungary (permit No. XVI/03047–2/2008).

FVB/N mice were purchased from Charles Rivers. A total of 40 animals (20 male and 20 female) were utilized for carcinogenesis experiments. A single dose (15 μg/g body weight) of DEN (N0258, Sigma-Aldrich, St. Louis, Missouri, US) was injected intraperitoneally at the age of 15 days. DEN concentration was chosen to be low enough to minimalize mutation occurrence and high enough for tumor formation within a year. A total of 14 mice served as age-matched untreated controls. Animals were terminated one year after DEN exposure by cervical dislocation in ether anesthesia. The body, lung and liver weight of mice were measured, and the number of macroscopically detectable tumors was recorded. Samples were fixed in 10% buffered formaldehyde and embedded in paraffin for histological analysis.

For generating subcutaneously maintained lung carcinoma, lung tumors were removed, cut into small pieces, washed in PBS and transplanted subcutaneously in another FVB/N mouse. The tumor was passaged when necessary. Samples were stored at − 70 °C. DNA was isolated from the primary tumor and tumor from the 14th passage.

### Immunohistochemistry

Formalin-fixed paraffin-embedded (FFPE) sections were dewaxed in xylene and ethanol then washed in distilled H_2_O. Antigen retrieval was carried out with citrate buffer (10 mM citric acid, 0.05% Tween 20, pH = 6.0) in a pressure cooker for 20 min. Slides were washed three times in phosphate buffered saline + 0.05% Tween 20 (PBST). Endogenous peroxidase block was applied for 10 min with 3% H_2_O_2_. After another washing procedure 5 *w*/*v*% bovine serum albumin (BSA)/PBS was used to block non-specific antibody binding sites. Primary antibodies were dissolved in 1 w/v% BSA/phosphate buffered saline (PBS) and applied for overnight at 4 °C. Primary antibodies were rabbit monoclonal anti-TTF1 (ab76013, Abcam, Cambridge, UK, dilution: 1:50) and rabbit polyclonal anti-CK7 (HPA007272, Atlas antibodies, Stockholm, Sweden, dilution: 1:100). The next day PBST wash was applied for 5 × 5 minutes. Secondary antibody was horse-radish peroxidase (HRP) conjugated anti-rabbit antibody (P0448, Dako, Glostrup, Denmark) applied for 1 h. After washing procedure signals were visualized with 3,3-diaminobenzidine tetrahydrochloride (DAB) substrate chromogen solution (Novolink Polymer Detection System, RE7150-K, Leica Biosystems, Wetzlar, Germany). Nuclei were stained by hematoxillin. The slides were scanned, and viewed with Pannoramic Viewer (3D Histech Ltd., Budapest, Hungary).

### Sanger sequencing

#### DNA isolation from frozen tissue and from FFPE sections

Frozen tumorous tissue was homogenized in liquid nitrogen and suspended with 400 μl lysis buffer (0.2 M NaCl, 0.02 M EDTA, 0.04 M Tris and 0.5% SDS) supplemented with 20 μl Proteinase K (10 mg/ml, Roche, Basel, Switzerland) and 2 μl β-mercaptoethanol.

On FFPE sections, tumorous area was marked under light microscope. Next, slides were dewaxed, and then rinsed in acetone and alcohol. After drying, the marked tumorous areas were removed by a scalpel and incubated in Tris-EDTA (10 mM Tris, 1 mM EDTA, pH = 7.4) buffer containing 2 mg/ml Proteinase K at 55 °C overnight with 300 rpm shaking to remove proteins.

The enzyme was heat-inactivated at 95 °C for 10 min followed by 15 min incubation on ice. Lysates were centrifuged at 13000 rpm for 15 min. The supernatants were kept and 50 μl, 5 M NaCl was added and incubated for another 15 min on ice. After centrifugation with 13,000 rpm for 10 min, 1 ml of ice-cold ethanol was added to each supernatant to precipitate DNA. The samples were centrifuged with 13,000 rpm for 10 min and the pellets were dried out with Savant AES 1000 SpeedVac system (Thermo Fischer Scientific, Waltham, MA). Pellets were dissolved in 50 μl TE buffer and DNA concentrations were measured with NanoDrop ND-1000 spectrophotometer (Thermo Fischer Scientific, Waltham, MA).

#### Polymerase chain reaction (PCR)

Primers designed for mouse EGFR exon 19, 21 and KRAS exon 2 are shown in Table 1.

**Table 1:** Primer sequences

Gene	Primer sequence
EGFR exon 19 forward	5’-CTGGATCCCAGAAGGTGAGA-3’
EGFR exon 19 reverse	5’-GGAAGCAAGATTGACCTTATGAA-3’
EGFR exon 21 forward	5’-TTGGCAGCCAGGAATGTACT-3’
EGFR exon 21 reverse	5’-GGCTGTCAGGAAAATGCTTC-3’
KRAS exon 2 forward	5’-TGTAAGGCCTGCTGAAAATG-3’
KRAS exon 2 reverse	5’-GCACGCAGACTGTAGAGCAG-3’

Reactions were performed in a total volume of 20 μl. ImmoMix Red 2× (BIO-25002, Bioline, London, UK) reaction-mix was used for the PCR. Twenty pmol of forward and reverse primer (Integrated DNA Technologies, Coralville, IA), MgCl_2_ (BIO-37026, Bioline, London, UK) and 50 ng DNA was added to each reaction. Thermal cycle parameters were as follows: 95 °C initial denaturation for 10 min followed by 40 cycles of denaturation at 95 °C for 40 s, annealing at appropriate temperature for 40 s (59 °C for KRAS exon 2, 56 °C for EGFR exon 19, 57 °C for EGFR exon 21 primers), elongation at 72 °C for 40 s. PCR was carried out using Veriti 96 Well Thermal Cycler (Thermo Fischer Scientific, Waltham, MA). PCR products were checked on a 2% agarose gel by electrophoresis.

#### Cycle sequencing and electrophoresis

For PCR product clean-up, ExoSap (Cat. no.: 78201, Affymetrix, Cleveland, OH) was applied following the instructions of the manufacturer. Cycle sequencing reactions were conducted using BigDye Terminator v3.1 Cycle Sequencing Kit (Cat. no.: 4337454, Thermo Fischer Scientific, Waltham, MA) as specified in the user guide. The subsequent cleaning step was performed with Nucleo-SEQ kit (Cat. no.: 740523, Macherey-Nagel, Düren, Germany) as described in the user manual. The samples were eluted in 20 μl formamide and denatured at 95 °C for 1 min. Capillary electrophoresis was carried out on a 3500 Series Genetic Analyzer (Thermo Fischer Scientific, Waltham, MA). Sequences were analyzed using BioEdit Sequence Alignment Editor (Ibis Biosciences, Carlsbad, CA).

### Western blot

Frozen tissues were homogenized and suspended with lysis buffer (containing: 20 mM Tris pH = 7.5, 150 mM NaCl, 2 mM EDTA, 0.05% Triton X-100, 0.5% Protease Inhibitor Cocktail (P8340, Sigma-Aldrich, St. Louis, MO), 2 mM Na_3_VO_4_ and 10 mM NaF). Protein concentrations were measured by Bradford method. For normal lung and primary tumors, lysates of 5 different specimens were pooled to generate one sample, and 3 different samples were prepared. Pooled primary tumor samples were individually analyzed checking for diversity (Additional file 1: Fig. S1). The selected proteins displayed quite homogenous distribution proving the applicability of pooled samples later on (Additional file 1: Fig. S1). For subcutaneous tumors, 1 tumor sample was run in each experiment. Thirty μg of total proteins were mixed with loading buffer containing β-mercaptoethanol and denatured at 99 °C for 5 min. Denatured samples were loaded onto a 10% SDS-polyacrylamide gel and separated for 40 min at 200 V. Proteins were transferred to a PVDF membrane with overnight blotting at 4 °C at constant 75 mA. Blotting efficiency was checked by Ponceau staining. Blocking procedure was carried out with 5% non-fat dry milk dissolved in Tris-buffered saline (TBS) applied for 1 h. Next, membranes were incubated overnight with primary antibodies. After washing with TBST (TBS + 0.05% Tween 20), appropriate secondary antibody dissolved in 1% non-fat dry milk (TBS) was applied for 1 h at room temperature. After washing, immunoreactions were visualized using SuperSignal West Pico Chemiluminescent Substrate kit (Cat. no.: 34078, Thermo Fischer Scientific, Waltham, MA). Bands were detected with Kodak Image Station 4000MM (Kodak, Rochester, NY). Western blots were run in 3 independent experiments. Antibodies with their appropriate dilutions used for Western blots are shown in Table 2.

**Table 2:** Antibodies and their specifications

Primary antibody	Manufacturer	Catalog number	Source	Dilution
Akt	Cell Signaling Technology, Danvers, MA	#4691	rabbit	1:1000
p-Akt (Thr308)	Cell Signaling Technology	#2965	rabbit	1:1000
p-Akt (Ser473)	Cell Signaling Technology	#4058	rabbit	1:1000
p-Erk 1/2	Cell Signaling Technology	#4370	rabbit	1:1000
p-GSK3 α/β	Cell Signaling Technology	#9331	rabbit	1:500
p-S6	Cell Signaling Technology	#2211	rabbit	1:1000
CDK4	Neomarkers, Fremont, California, US	#MS-616	mouse	1:250
PCNA	Atlas antibodies, Stockholm, Sweden	HPA030522	rabbit	1:1000
p-Rb S780	Cell Signaling Technology	#9307	rabbit	1:250
β-actin	Sigma-Aldrich, St. Louis, MO	A2228	mouse	1:5000

## Results

### Activity of DEN to induce lung cancer

We found DEN to be a potent lung carcinogen in the FVB/N mouse strain. Out of 39 mice, 28 developed macroscopic lung tumors. Six of them had multiple neoplasia (Table 3). The tumor prevalence between the genders showed only minor differences and no differences in lung mass was observed (Table 3). Histologically, tumors appeared to be papillary carcinomas, their morphology was very similar to that observed in human disease. Most tumors were moderately or well-differentiated, a few showed poorly differentiated appearance. Multiple neoplasias often had mixed phenotype.

**Table 3:** Tumor prevalence in FVB/N mice induced by DEN

Gender	Total No.	Animals with macroscopic lung tumors	Average lung mass	Average lung mass/ body mass (%)
Male	19	15	0.24 g	0.78
Female	20	13	0.23 g	0.78

Out of 14 untreated control only two mice developed spontaneous lung adenocarcinoma (Table 4). Only males were observed in the control group because there was no significant difference in the lung tumor development between the males and females in the literature [18]. The two tumors appeared to be poorly differentiated papillary carcinomas; however the low number prevents its comparison to DEN-induced tumors.

**Table 4:** Tumor prevalence in control, untreated FVB/N mice

Total No.	Animals with macroscopic lung tumors	Average lung mass	Average lung mass/ body mass (%)
14	2	0.15 g	0.5

### Histochemical markers of lung adenocarcinoma

**Fig. 1:**
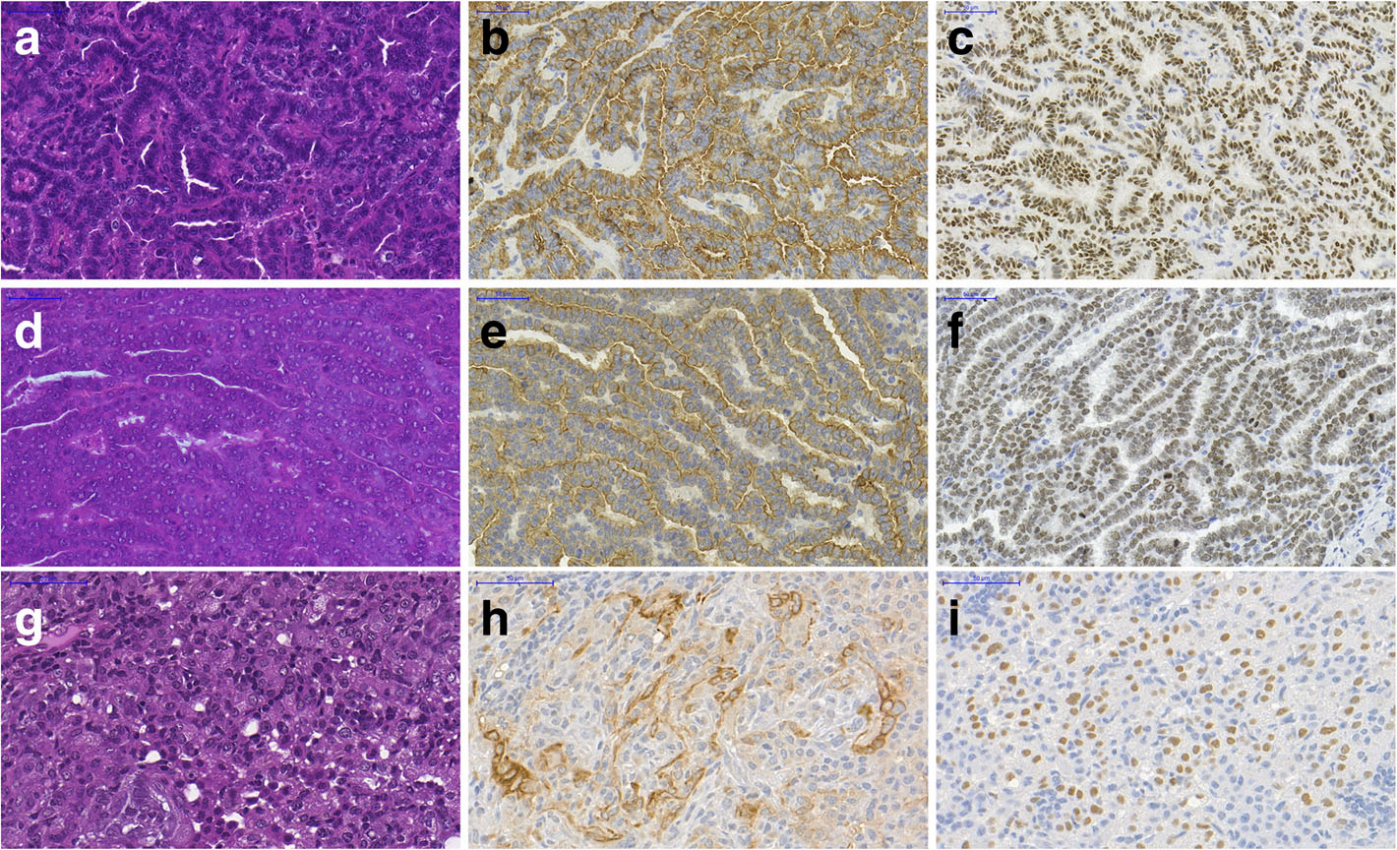
Histological analysis of primary, subcutaneous and spontaneous lung adenocarcinoma. (**a**-**c**) Primary tumor; (**d**-**f**) Subcutaneous tumor; (**g**-**i**) Spontaneous tumor, (**a**,**d**,**g**) Hematoxylin and eosin staining. Immunostaining of cytokeratin-7 (**b**,**e**,**h**) and TTF1 (**c**,**f**,**i**). Primary and subcutaneous tumors are from different animals. Scale bar = 50 μm

With immunostaining all the 28 tumorous tissues showed high level of CK7 and TTF1 expression which confirmed the lung adenocarcinoma diagnosis (Fig. 1). The spontaneous lung tumors also showed CK7 and TTF1 positivity.

### KRAS and EGFR sequencing

**Fig. 2:**
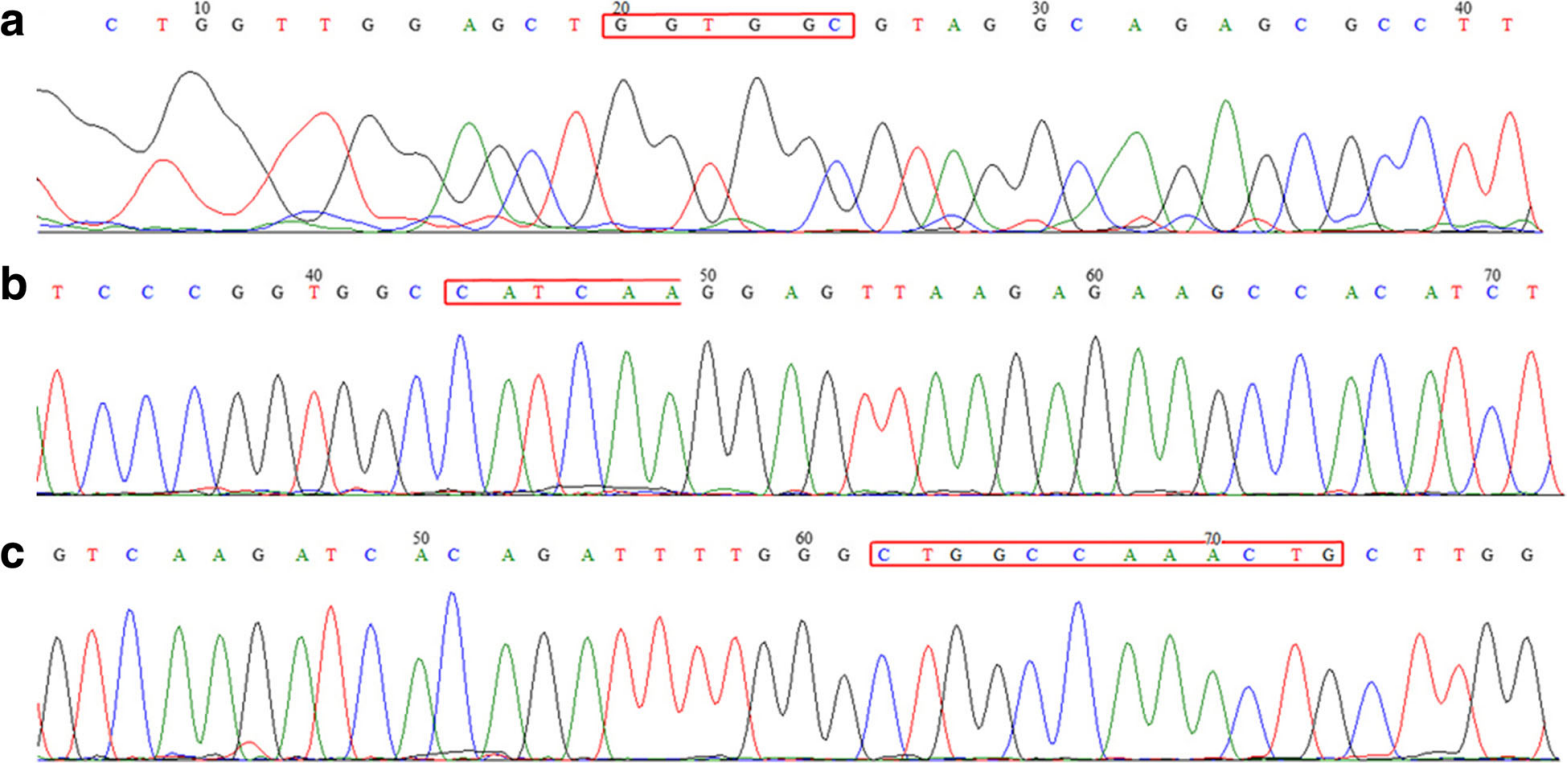
Mutation analysis by Sanger sequencing. (**a**) KRAS sequence, (**b**) EGFR exon 19 sequence, (**c**) EGFR exon 21 sequence; Regions of potential mutations are marked in the sequences

KRAS and EGFR mutation hot spots were analyzed in all tumorous samples including spontaneous tumors. No mutations were found in KRAS exon 2 and in EGFR exons 19 and 21 (Fig. 2).

### Alterations in signaling pathways

**Fig. 3:**
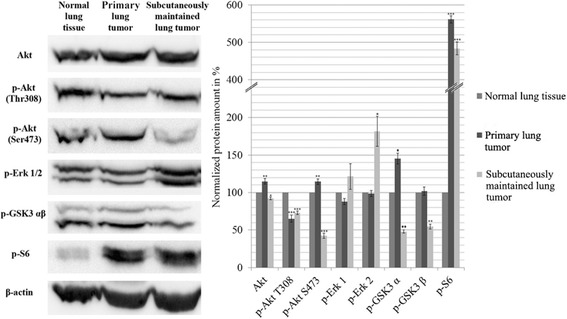
Western blot analysis of the main signaling pathways. The data are the mean ± SD of 3 experiments, *P < 0.05; **P < 0.01 and ***P < 0.001. Blots are separate images

Akt/mTOR, ERK pathways and G1/S restriction point were analyzed by Western blot technique. We compared normal FVB/N lung tissue with the DEN induced primary adenocarcinoma and with the subcutaneously maintained lung tumor. The amount of Akt phosphorylated at Thr308 and Ser473 significantly decreased in the subcutaneously maintained sample, and p-Akt Thr308 in the DEN induced primary tumor showed similar tendency compared to the normal lung tissue, whereas Ser473 remained unchanged in the primary tumor. On the other hand p-S6 increased ~ 5-fold in both tumor samples. While p-Erk1/2 in the primary tumor did not differ from the control, it was greatly increased (20% and 80% respectively) in the subcutaneously maintained tumor compared to the normal tissue. The phosphorylation of GSK3β decreased in the subcutaneous adenocarcinoma, while it remained unchanged in the primary tumor (Fig. 3).

**Fig. 4:**
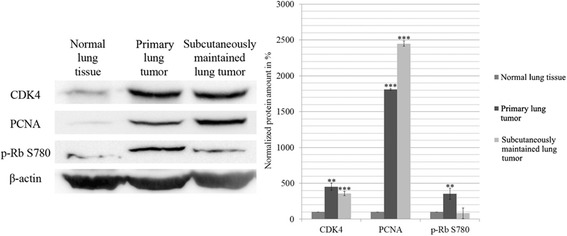
Western blot analysis of the main cell cycle proteins. The data are the mean ± SD of 3 experiments, *P < 0.05; **P < 0.01 and ***P < 0.001. Blots are separate images

Regarding cell cycle regulation, the level of CDK4 responsible for retinoblastoma phosphorylation in G1-S transition, increased with ~ 4-fold in both tumor samples. Phospho-Rb S780 increased significantly in the primary tumor, but interestingly decreased in the subcutaneous tumor. The S-phase marker PCNA showed a remarkable ~ 18-fold elevation in the primary tumor and ~ 25-fold in the subcutaneous tumor compared to the normal tissue indicating their increased proliferation. (Fig. 4).

## Discussion

DEN is a commonly used agent to induce liver cancer [15, 19]. In addition, some literature data indicates that chemical carcinogens, such as DEN, can be a potent lung carcinogen in strains where the frequency of the lung tumors was already higher than any other cancer [13, 14, 17]. In the literature DEN was used in a concentration of 50 mg/kg to induce lung tumor and only KRAS was analyzed, which was found to be 80% mutated. We applied a single dose of 15 μg/g intraperitoneally, hypothetically low enough to minimize mutation occurrence and ideally avoid mutations in KRAS and EGFR genes. In our cases neither primary nor subcutaneously maintained tumors harbored mutations in EGFR exon 19, 21 or KRAS exon 2. These data may indicate a dose dependent mutation forming effect, but a more probable hypothesis is that the A/J mouse strain is more susceptible to lung cancer with mutant KRAS, simply because of it has different genetic background compared to that of FVB/N. In human adenocarcinomas the frequency of EGFR mutations is estimated between 15 and 45%, whereas KRAS mutation was detected in 20% of the cases depending on the geographical region [20]. However, our model could represent the portion of human adenocarcinomas negative for KRAS and EGFR mutations.

FVB/N mice is a well-known and described mouse strain, which is more susceptible to develop spontaneous lung cancer than any other tumor and the ratio of liver tumors are lower than in other strains [18]. This susceptibility can explain why DEN induced tumors predominantly in the lung, and very few in the liver until the 1st year endpoint. DEN might speed up the already existing susceptibility for lung cancer formation which theory is in harmony with other literatures [14]. Regarding morphology, tumors in our model appeared to be papillary carcinomas, all TTF-1 and cytokeratin-7 positive analogous to human lung cancers.

The increased p-S6 indicates a more active mTOR signaling which leads to cell proliferation [21]. Interestingly the amount of both p-Akt is decreased in the subcutaneously maintained tumor and a reduction can also be seen in the level of p-Akt Ser473 in the primary tumor, as well. Phosphorylation of Erk1/2 increased in the subcutaneous tumor which can be one of the mechanism that results in active mTOR pathway [22]. The decreased amount of p-GSK3 correlates well with the low level of p-Akt in all the samples.

The proliferation stimuli results in a constantly working cell cycle which can be seen in the two tumorous samples. CDK4 and the S phase marker PCNA are greatly increased in both tumors compared to the normal lung sample [23, 24]. Increased p-Rb S780 in the primary tumor also confirms this, but interestingly it decreased in the subcutaneous tumor. This could be explained by the hyperphosphorylation of Rb which can lead to its degradation [25]. It is also possible that Rb protein was lost due to deleterious mutation [26].

These results prove that the FVB/N mouse strain can be used for lung cancer experiments, because chemical carcinogens speed-up its already accelerated lung tumorigenesis resulting adenocarcinomas. In addition, our model is unique as it can better represent human lung adenocarcinoma cases with no common KRAS and EGFR mutations. Thus we believe that this well-characterized model with unraveled signaling pathways has a value in both academic and practical use.

## Conclusions

We found diethylnitrosamine as a potent chemical substance to induce lung adenocarcinoma in FVB/N mouse strain. The tumor was positive for CK7 and TTF1. We found no mutations in KRAS exon 2 and EGFR exon 19 and 21. The proliferation of the tumors is driven by the MAPK and mTOR pathways ending up with the stimulation of cell cycle at the G1/S restriction point. The model could be a useful tool in lung cancer research targeting KRAS and EGFR negative tumors.

## Additional file

Additional file 1: Figure S1. Western blot analysis of pooled tumor samples. (A) Image of Western blot run loaded with individual primary tumor samples. (B-C) Quantification of various protein amounts. (D) Average of protein levels measured in individual tumors (data are expressed as mean ± SD). (JPEG 522 kb)

## Abbreviations

BSA: bovine serum albumin
CDK: cyclin-dependent kinase
CK7: cytokeratin-7
DAB: 3,3-diaminobenzidine
DEN: diethylnitrosamine
EGFR: epidermal growth factor receptor
FFPE: Formalin-fixed paraffin-embedded
HRP: horse-radish peroxidase
KRAS: Kirsten rat sarcoma
mTOR: mammalian target of rapamycin
NSCLC: non-small cell lung cancer
PBS: phosphate-buffered saline
PCNA: Proliferating cell nuclear antigen
PCR: polymerase chain reaction
Rb: retinoblastoma
SCLC: small cell lung cancer
TBS: tris-buffered saline
TTF1: thyroid transcription factor-1
