# Supplementary figures and images for: Diethylnitrosamine induces lung adenocarcinoma in FVB/N mouse
Source: BMC Cancer. 2018 Feb 7;18:157. doi: 10.1186/s12885-018-4068-4 (PMC5803903; doi:10.1186/s12885-018-4068-4)

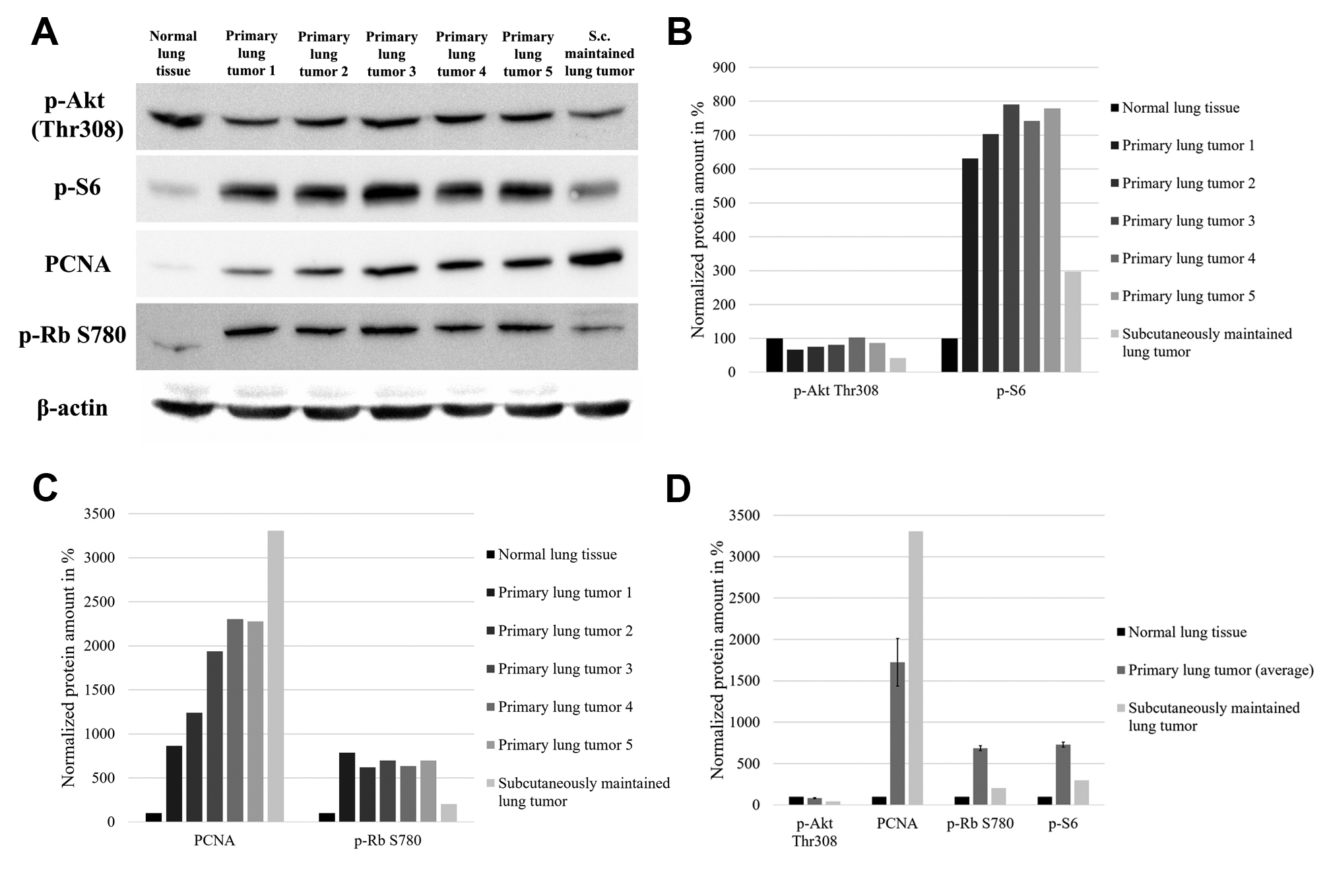

Supplement: Additional file 1: Figure S1. — Western blot analysis of pooled tumor samples. (A) Image of Western blot run loaded with individual primary tumor samples. (B-C) Quantification of various protein amounts. (D) Average of protein levels measured in individual tumors (data are expressed as mean ± SD). (JPEG 522 kb) [file 12885_2018_4068_MOESM1_ESM.jpg]
